# Supplementary material for: No evidence that Chinese playtime mandates reduced heavy gaming in one segment of the video games industry
Source: Nat Hum Behav. 2023 Aug 10;7(10):1753–66. doi: 10.1038/s41562-023-01669-8 (PMC10593605; doi:10.1038/s41562-023-01669-8)
Supplement: Supplementary file 1 — Supplementary Methods including Table 1 and Figs. 1 and 2. [file 41562_2023_1669_MOESM1_ESM.pdf]

nature human behaviour

Article https://doi.org/10.1038/s41562-023-01669-8

No evidence that Chinese playtime mandates reduced heavy gaming in one segment of the video games industry

In the format provided by the authors and unedited

Chinese playtime mandates did not reduce heavy gaming in one segment of the video games industry: Supplementary event study analysis.

In this supplementary subsection, we report a series of additional sensitivity analyses using event study paradigms.

Method

A novel dataset was used for the purposes of these supplementary analyses. This is reported below as dataset 6.

Dataset 6 was created for the purpose of sensitivity analysis following peer review. It contains exactly 2,000,000 datapoints: 1,000,000 random observations drawn from China during the 22 weeks under analysis and 1,000,000 random control observations drawn from other countries within the East Asian cultural sphere (South Korea, Taiwan, Hong Kong, Japan, and Vietnam) during the 22 weeks under observation. Each observation represents the interactions of a specific player account during a specific week, and contains the following information:

1. Total hours played during that week.
2. Whether playtime was heavy or not heavy for that week (as defined elsewhere in this paper).
3. The country that playtime took place in.
4. The place within that country that playtime took place in, where places typically represent cities or analogous municipal subdivisions within a country. Representative places include Shanghai, Chengdu, Beijing, Suzhou, and Shenzhen.

Analytical Approach

Our analytical approach here estimates changes in weekly outcomes for observations occurring within China relative to a set of control observations before and after the implementation of playtime mandates. We assess this using an event study model that estimates outcomes while controlling for fixed differences across places and overall trends over time. We estimate:

$$Y_{ipt} = R_p \times \sum_{\substack{w=-11 \\ w\neq -1}}^{10} \beta_w I(t - t_p^* = w) + \beta_t + \beta_p + \epsilon_{ipt}$$

Our data are constructed at the account (i) by week (t) level, with data being recorded each week in a specific place (p). In this equation the variable R_p equals '1' if an individual account (i) was observed within a place that enacted playtime mandates (i.e. a place within China). Indicator variables $I(t - t_p^* = w)$ measure time relative to the implementation of playtime mandates, and are zero for all periods in non-Chinese places. The omitted category is $w = -1$, the week prior to mandates coming into effect. Therefore, each estimate of β_w provides the difference in outcomes in Chinese places relative to non-Chinese places during week w, as measured from the reference week immediately prior to mandates coming into effect. β_t and β_p estimate fixed effects of week and place respectively. We estimate this model with a fixed-effects OLS model using the *feols* (0.8.4) package in R, and report standard errors clustered at the place level.

In addition to event study analysis, we also present difference-in-difference estimates as a summary of the effect across all post-mandate weeks. These are estimated using the same equation except that event study indicators are replaced with a single variable denoting a Chinese place during the post-mandate period. This indicator turns on starting when $w = 0$.

In order to be as robust as possible two models are built. Our first model assesses the relationship between playtime mandates and likelihood of heavy play. If a week's playtime was heavy our outcome is coded '1', and if it is not heavy our outcome is coded '0'. Our second model assesses the relationship between playtime mandates and the amount of playtime that individuals engage in per week. The outcome variable in this case is the total hours that an account was recorded as playing during that week.

Results

Results are presented in Table 1 and Figures 1-2 below. Firstly, these results indicate that the presence of playtime mandates was not associated with lessened likelihood of heavy play amongst individual accounts. Difference-in-difference analysis fail to show any significant change in heavy play rates amongst accounts within China when compared to those from elsewhere in the East Asian cultural sphere (p=0.52). Similarly, results also indicate that the presence of playtime mandates was not associated with changes in total playtime amongst individual accounts. Again, difference-in-difference analysis are unable to show a change to this variable amongst accounts within China when compared to those from elsewhere in the East Asian cultural sphere (p=0.70).

	Played heavily			Total hours played		
	Estimate	Std. Error	p-value	Estimate	Std. Error	p-value
**Difference-in-differences model**						
	0.00020	0.00031	0.5258	0.01114	0.02884	0.6993
**Event study model**						
Week -11	-0.00234*	0.00091	0.0103	-0.05843	0.06771	0.3882
Week -10	-0.00164	0.00097	0.0894	-0.02377	0.06713	0.7232
Week -9	-0.00226*	0.00090	0.0125	-0.05277	0.06753	0.4346
Week -8	-0.00084	0.00088	0.3395	0.00281	0.06525	0.9656
Week -7	-0.00222*	0.00096	0.0206	-0.20173**	0.06511	0.0020
Week -6	-0.00273*	0.00115	0.0182	-0.01999	0.08034	0.8035
Week -5	-0.00402***	0.00093	<0.0001	-0.34495***	0.06106	<0.0001
Week -4	-0.00310**	0.00104	0.0029	-0.22870**	0.06979	0.0011
Week -3	-0.00167	0.00101	0.1008	-0.11856	0.06480	0.0674
Week -2	-0.00277**	0.00085	0.0012	-0.21846***	0.05755	0.0001
Week -1 (omitted)	-	-				
Week 0	-0.00190*	0.00091	0.0359	-0.07087	0.06053	0.2418
Week 1	-0.00113	0.00091	0.2136	0.01668	0.06560	0.7993
Week 2	-0.00022	0.00090	0.8051	-0.04710	0.07372	0.5229
Week 3	-0.00050	0.00099	0.6163	0.02506	0.06654	0.7065
Week 4	-0.00157	0.00098	0.1096	-0.07156	0.07619	0.3477
Week 5	-0.00214*	0.00099	0.0314	-0.06149	0.06498	0.3441
Week 6	-0.00201*	0.00091	0.0272	-0.11810*	0.05963	0.0477
Week 7	-0.00231**	0.00084	0.0060	-0.07947	0.06305	0.2076
Week 8	-0.00317**	0.00099	0.0014	-0.16841*	0.06696	0.0119
Week 9	-0.00332***	0.00090	0.0002	-0.23707***	0.06553	0.0003
Week 10	-0.00285**	0.00087	0.0010	-0.29496***	0.06566	<0.0001

Table 1: Event study coefficients associated with this study. Significant levels: *p<0.05, **p<0.01, ***p<0.001. All coefficients are calculated via fixed-effects OLS estimation using the analytical approach outlined above. P-values are two-tailed. No adjustments were made for multiple comparisons.

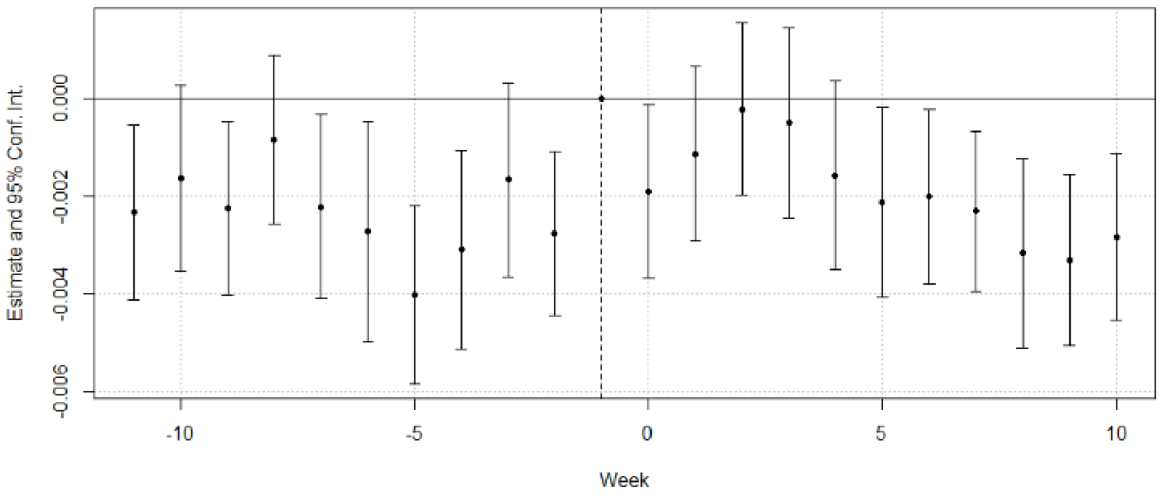

Figure 1: Relationship between the presence of playtime mandates and heavy play. Coefficients are reported in relation to a reference week, which is the week prior to mandates coming into effect (week -1). Data consist of n=2,000,000 weekly sessions observed over n=22 weeks. Confidence intervals are calculated with the *coefplot* function from the *fixest* package in R (v0.10.4) using standard errors clustered at the place level.

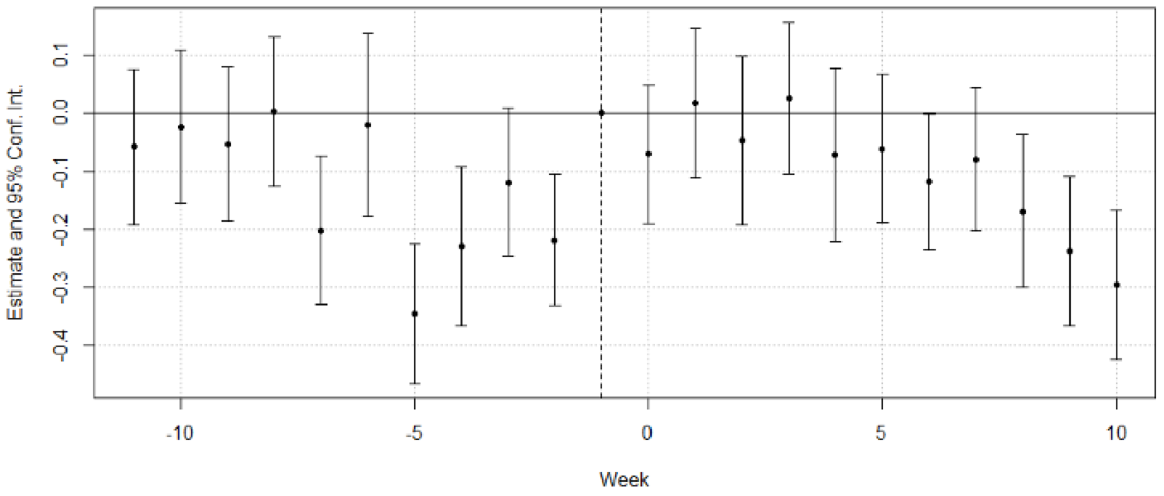

Figure 2: Relationship between the presence of playtime mandates and total playtime per account. Coefficients are reported in relation to a reference week, which is the week prior to mandates coming into effect (week -1). Data consist of n=2,000,000 weekly sessions observed over n=22 weeks. Confidence intervals are calculated with the *coefplot* function from the *fixest* package in R (v0.10.4) using standard errors clustered at the place level.
