# Supplementary material for: No evidence that Chinese playtime mandates reduced heavy gaming in one segment of the video games industry
Source: Nat Hum Behav. 2023 Aug 10;7(10):1753–66. doi: 10.1038/s41562-023-01669-8 (PMC10593605; doi:10.1038/s41562-023-01669-8)
Supplement: Supplementary file 2 — Reporting Summary [file 41562_2023_1669_MOESM2_ESM.pdf]

nature portfolio

Corresponding author(s): David Zendle
Last updated by author(s): 04/06/2023

Reporting Summary

Nature Portfolio wishes to improve the reproducibility of the work that we publish. This form provides structure for consistency and transparency in reporting. For further information on Nature Portfolio policies, see our Editorial Policies and the Editorial Policy Checklist.

Statistics

For all statistical analyses, confirm that the following items are present in the figure legend, table legend, main text, or Methods section.

n/a	Confirmed	
☐	☒	The exact sample size (*n*) for each experimental group/condition, given as a discrete number and unit of measurement
☐	☒	A statement on whether measurements were taken from distinct samples or whether the same sample was measured repeatedly
☐	☒	The statistical test(s) used AND whether they are one- or two-sided<br>*Only common tests should be described solely by name; describe more complex techniques in the Methods section.*
☐	☒	A description of all covariates tested
☐	☒	A description of any assumptions or corrections, such as tests of normality and adjustment for multiple comparisons
☐	☒	A full description of the statistical parameters including central tendency (e.g. means) or other basic estimates (e.g. regression coefficient) AND variation (e.g. standard deviation) or associated estimates of uncertainty (e.g. confidence intervals)
☐	☒	For null hypothesis testing, the test statistic (e.g. *F*, *t*, *r*) with confidence intervals, effect sizes, degrees of freedom and *P* value noted<br>*Give P values as exact values whenever suitable.*
☒	☐	For Bayesian analysis, information on the choice of priors and Markov chain Monte Carlo settings
☒	☐	For hierarchical and complex designs, identification of the appropriate level for tests and full reporting of outcomes
☐	☒	Estimates of effect sizes (e.g. Cohen's *d*, Pearson's *r*), indicating how they were calculated

Our web collection on statistics for biologists contains articles on many of the points above.

Software and code

Policy information about availability of computer code

Data collection	Google BigQuery (no relevant versioning that we are aware of) was used to collect the data in this study. This is a cloud-based serverless data warehouse with an associated SQL-like query language.
Data analysis	Data analysis was conducted using R (v.4.0.2). An OSF repository containing relevant code is available at: doi.org/10.17605/OSF.IO/AUH2K

For manuscripts utilizing custom algorithms or software that are central to the research but not yet described in published literature, software must be made available to editors and reviewers. We strongly encourage code deposition in a community repository (e.g. GitHub). See the Nature Portfolio guidelines for submitting code & software for further information.

Data

Policy information about availability of data

All manuscripts must include a data availability statement. This statement should provide the following information, where applicable:

- Accession codes, unique identifiers, or web links for publicly available datasets
- A description of any restrictions on data availability
- For clinical datasets or third party data, please ensure that the statement adheres to our policy

Data for this study were provided by Unity Technologies under a data sharing agreement between this organization and the lead author's host institution (the University of York). The data under analysis here cannot be made available to external scholars. This restriction on public access is grounded in the terms of the data

sharing agreement formed between Unity Technologies and the lead author's host institution and is linked to the commercially sensitive nature of these large-scale data.

Research involving human participants, their data, or biological material

Policy information about studies with human participants or human data. See also policy information about sex, gender (identity/presentation), and sexual orientation and race, ethnicity and racism.

Reporting on sex and gender	There were no participants in this research - it was conducted purely on anonymised secondary data.
Reporting on race, ethnicity, or other socially relevant groupings	There were no participants in this research - it was conducted purely on anonymised secondary data.
Population characteristics	There were no participants in this research - it was conducted purely on anonymised secondary data.
Recruitment	There were no participants in this research - it was conducted purely on anonymised secondary data.
Ethics oversight	This study protocol was approved by the Physical Sciences Ethics Committee at the University of York.

Note that full information on the approval of the study protocol must also be provided in the manuscript.

Field-specific reporting

Please select the one below that is the best fit for your research. If you are not sure, read the appropriate sections before making your selection.

☐ Life sciences ☒ Behavioural & social sciences ☐ Ecological, evolutionary & environmental sciences

For a reference copy of the document with all sections, see nature.com/documents/nr-reporting-summary-flat.pdf

Behavioural & social sciences study design

All studies must disclose on these points even when the disclosure is negative.

Study description

This is a quantitative observational study, involving longitudinal measurements at both population and within-participant levels. In brief, in November 2019 the People's Republic of China (PRC) enacted a novel policy which was designed to limit the amount of playtime that individuals were able to engage in within video games. In this study, we test the impact of this policy on heavy play.

We test this via two primary preregistered analyses: In a first analysis, we investigate whether the overall prevalence of heavy play decreases week-on-week after regulation is put in place.

In a second, within-participant, analysis, we take a sample of accounts who were active both before and after regulation, and investigate whether the presence of playtime regulations can explain substantial variance in the likelihood of these accounts playing heavily on any given week.

Several further sensitivity analyses are undertaken, with unique datasets underpinning these. These data range from counts of the number of video game installs in PRC during the period in question; to sensitivity analyses over the number of hours played during the period in question (Rather than the prevalence of heavy play); to an analysis of an analogous period during 2021.

Research sample

The primary sample under analysis are 2,486,192,234 unique profiles of gamers drawn from games that utilise Unity Analytics, and which were geolocated in PRC during the 22 week period under study. This sample is unable to represent the entire games market in PRC (many games are not developed using Unity, and patterns of play may vary in these games). However, it is able to represent the population of gamers who engage with Unity products, which is substantial (over 7bn hours of playtime within this dataset alone) and of real-world importance.

For the second, within-participants analysis, we subsampled 10,000 accounts from within this dataset which were active during all 22 weeks under study. This data is thus able to represent the subset of players who played games made using Unity Analytics, and who played consistently during this period within the PRC.

The source for all datasets and analyses described above were Unity Technologies' internal data lakes, which anonymously record the presence of play sessions in games which implement Unity Analytics.

Sampling strategy

Data in our primary dataset are not subsampled at all: They represent every account engaging with a product that implements Unity Analytics within the PRC during the period of interest.

For our within-participants analysis, our base data consisted of all individual gamer profiles in our data whose IP addresses identified them as being from China for every play session prior to 1st November 2019; and who engaged in play at least once during each of the 22 weeks under analysis here (i.e. 16th August 2019 to 16th January 2020). We randomly selected 10,000 accounts from this base to form our dataset. The reason for the subsampling was computational tractability: The modelling approach selected for this study would have taken an infeasibly large amount of memory and time if the sample was significantly larger.

Data collection	The data used in this study is data from Unity Analytics that is pseudonymised by way of a token unique to each player of each individual game - no players are traceable across games, nor are players identifiable from this data. Unity technologies collect and store user-generated data using a plug-in for its engine known as Unity Analytics. The researcher was not blinded to experimental conditions and/or study hypotheses when collecting this data.
Timing	The primary data are drawn from a 22-week period surrounding Nov 1st 2019 - the 11 weeks (i.e. 7-day periods) immerdiately preceding and following this date.
Data exclusions	The subset of Unity data used for this study was generated by selecting all daily playtime logs which (a) were identified as taking place within the PRC; (b) took place on a date equal to or greater than 16th August 2019; (c) Took place on a date equal to or less than 16th January 2020. A final exclusion criterion was that all products which implemented Unity Analytics but whose metadata identified them as not being a game were not included in this study.
Non-participation	No participants were involved in this study.
Randomization	This is an observational study taking place over historic secondary data, and thus typical randomisation methods (e.g. assigning participants to specific conditions) are not applicable here. The only case in which randomisation could be said to be employed during this study was the selection of 10,000 random accounts for the analysis of within-person effects, as outlined above.

Reporting for specific materials, systems and methods

We require information from authors about some types of materials, experimental systems and methods used in many studies. Here, indicate whether each material, system or method listed is relevant to your study. If you are not sure if a list item applies to your research, read the appropriate section before selecting a response.

Materials & experimental systems

n/a	Involved in the study
☒	☐ Antibodies
☒	☐ Eukaryotic cell lines
☒	☐ Palaeontology and archaeology
☒	☐ Animals and other organisms
☒	☐ Clinical data
☒	☐ Dual use research of concern
☒	☐ Plants

Methods

n/a	Involved in the study
☒	☐ ChIP-seq
☒	☐ Flow cytometry
☒	☐ MRI-based neuroimaging
